# Dichotomy in the *NRT* Gene Families of Dicots and Grass Species

**DOI:** 10.1371/journal.pone.0015289

**Published:** 2010-12-06

**Authors:** Darren Plett, John Toubia, Trevor Garnett, Mark Tester, Brent N. Kaiser, Ute Baumann

**Affiliations:** 1 Australian Centre for Plant Functional Genomics, Waite Research Institute, University of Adelaide, Adelaide, South Australia, Australia; 2 School of Agriculture, Food and Wine, Waite Research Institute, University of Adelaide, Adelaide, South Australia, Australia; Kyushu Institute of Technology, Japan

## Abstract

A large proportion of the nitrate (NO_3_^−^) acquired by plants from soil is actively transported via members of the *NRT* families of NO_3_^−^ transporters. In Arabidopsis, the *NRT1* family has eight functionally characterised members and predominantly comprises low-affinity transporters; the *NRT2* family contains seven members which appear to be high-affinity transporters; and there are two *NRT3* (*NAR2*) family members which are known to participate in high-affinity transport. A modified reciprocal best hit (RBH) approach was used to identify putative orthologues of the Arabidopsis *NRT* genes in the four fully sequenced grass genomes (maize, rice, sorghum, *Brachypodium*). We also included the poplar genome in our analysis to establish whether differences between Arabidopsis and the grasses may be generally applicable to monocots and dicots. Our analysis reveals fundamental differences between Arabidopsis and the grass species in the gene number and family structure of all three families of NRT transporters. All grass species possessed additional *NRT1.1* orthologues and appear to lack *NRT1.6/NRT1.7* orthologues. There is significant separation in the *NRT2* phylogenetic tree between *NRT2* genes from dicots and grass species. This indicates that determination of function of *NRT2* genes in grass species will not be possible in cereals based simply on sequence homology to functionally characterised Arabidopsis *NRT2* genes and that proper functional analysis will be required. Arabidopsis has a unique *NRT3.2* gene which may be a fusion of the *NRT3.1* and *NRT3.2* genes present in all other species examined here. This work provides a framework for future analysis of NO_3_^−^ transporters and NO_3_^−^ transport in grass crop species.

## Introduction

Nitrogen use efficiency (NUE) in plants is determined by the efficiency with which the plant acquires and uses nitrogen. Nitrate (NO_3_^−^) is the primary nitrogen source for most plants in agricultural soils; cereal crops, however, access only 33–50% on average of the NO_3_^−^ applied to the soil by farmers [1], [2]. In order to improve this efficiency a more complete understanding of the transport of NO_3_^−^ from the soil to the plant and within the plant itself is required. An important first step towards improving the NO_3_^−^ uptake capacity and the NUE of crop plants would be characterisation of the transporters responsible for NO_3_^−^ transport. Either the expression of the relevant genes or else the function of the proteins encoded by the genes could then be manipulated through traditional plant breeding or genetic engineering in order to improve NO_3_^−^ uptake characteristics. With this goal in mind, the aim of this research was to identify the NO_3_^−^ transporters in grass species.

The transport of NO_3_^−^ and the transporters involved in this process have best been characterised in Arabidopsis due both to its amenability to physiological analyses and availability of genetic resources. The transport of NO_3_^−^ is mediated largely by members of the *NRT* gene families which have recently been reviewed [3]. The Arabidopsis genome contains 53 *NRT1*(*PTR*) family genes [3]. Only AtNRT1.1 to AtNRT1.8, however, have functional analyses indicating that these proteins do indeed transport NO_3_^−^. The *NRT1* family comprises predominantly low-affinity NO_3_^−^ transporters, with the exception of AtNRT1.1 which appears to mediate dual-affinity NO_3_^−^ transport [4], [5] based on phosphorylation status of the amino acid residue T101 [6]. A recent study indicated that AtNRT1.1 may also function as an NO_3_^−^ sensor [7]. The expression of *AtNRT1.2* is constitutive and located predominantly in the root epidermis indicating that the encoded transporter may also be involved in NO_3_^−^ uptake from the soil [8]. The expression of *AtNRT1.3* in roots is repressed by exposure to NO_3_^−^ and is induced by NO_3_^−^ deprivation; its functional role, however, remains less clear [9], [10]. *AtNRT1.4* is expressed primarily in the leaf petiole and appears to be involved in NO_3_^−^ storage [11]. AtNRT1.5 appears to mediate NO_3_^−^ efflux and to have a role in the loading of NO_3_^−^ into the xylem for transport to the shoot [12]. AtNRT1.6 is involved in transporting NO_3_^−^ from maternal tissue to developing embryos [13]. AtNRT1.7 has been identified as playing role in the remobilisation of NO_3_^−^ from older to younger leaves through facilitating phloem loading [14]. Very recently Li et al [15] have shown that AtNRT1.8 is responsible for retrieving NO_3_^−^ from the xylem parenchyma in the roots and shoots, thus working synergistically with AtNRT1.5 to control long-distance NO_3_^−^ transport.

The *NRT2* family are high-affinity NO_3_^−^ transporters comprising NO_3_^−^ inducible and constitutively expressed members [3]. The best characterised members are *AtNRT2.1* and *AtNRT2.2*, which are located next to each other on chromosome 1 and appear to encode proteins with similar function [16]. AtNRT2.1 seems to be more crucial for NO_3_^−^ influx and is expressed in the root cortex and epidermis [17]. Both *AtNRT2.1* and *AtNRT2.2*, however, are inducible by provision of NO_3_^−^ to NO_3_^−^ starved plants [10], and compensate for one another in that expression of either increases when the other is reduced [16]. Little is known about *AtNRT2.3* other than its expression may increase and decrease in cycles over the life cycle in the roots and shoots (mostly shoot) [9], [10]. *AtNRT2.4* is expressed predominantly in the root, and expression appears to decrease following exposure of plants to NO_3_^−^ [9], [10]. Similarly, *AtNRT2.5* is expressed in the root and shoot (mostly root) and is repressed by the provision of NO_3_^−^ [9], [10]. The expression of *AtNRT2.6* remains relatively unchanged in roots and shoots (mostly root) following exposure of plants to NO_3_^−^ [9], [10]. AtNRT2.7 appears to have a role in storage of NO_3_^−^ in seeds [18].

The *NRT3* genes in Arabidopsis play a role in NO_3_^−^ transport through regulating the activity of *NRT2* genes, but are not themselves transporters [19], [20]. The two *NRT3* genes appear to be closely related, but NRT3.1 (NAR2.1) appears to play the more significant role in high-affinity NO_3_^−^ uptake [20]. Although the recent annotation of the Arabidopsis genome has indicated that *AtNRT3.2* gene is larger than originally published [20], the significance of this fact is unknown (http://www.arabidopsis.org/).

Here, bioinformatic analyses are presented of the *NRT1*, *NRT2* and *NRT3* gene families in the four fully sequenced grass genomes of rice [21], [22], [23], *Brachypodium* [24], maize [25] and sorghum [26]. Also included is an analysis of poplar as a further fully sequenced dicot species [27], with the purpose of strengthening observations made on the dichotomy between Arabidopsis and the grass species. The analyses were limited to fully sequenced genomes to ensure completeness and to increase the utility of the work for informing further research into NO_3_^−^ transporters in grass species. The evolution from a common ancestor of the four species studied is such that they provide a good indication of the diversity of genomes within the grass species; maize and sorghum are the most closely related, having diverged an estimated 12 million years ago [24]. Also, in order to clarify past identification of the relevant genes and to provide a standardised framework for future researchers, a nomenclature for the grass *NRT* genes is presented.

## Results

A commonly accepted, extensively used and well documented method for the determination of genes that share a common evolutionary ancestor (orthologues, paralogues) across two genomes is the reciprocal best hit (RBH) [28], [29]. The RBH approach assumes that orthologous sequences hit to each other as the best scoring hit in a pairwise search between two genomes. There are however, limitations in the RBH method; it does not take parology into account and the highest scoring protein reported by BLAST is often not the nearest phylogenetic neighbour (high false negative rate) [30]. In this study we elected to use the RBH method over other orthology detection strategies due to its high stringency and success in identifying orthologues with a low false positive rate [31], [32]. To overcome the above mentioned shortcomings, a modified RBH, not restricted to the top hits alone and extended to include steps of refinement and validation was used (see Materials and Methods).

### *NRT1* family

As the genetic sequence(s) or protein motif(s) that separate the *NRT1* genes from the *PTR* genes (the protein products of which transport peptides) are unknown, the analysis here was limited to determining putative grass orthologues of eight functionally characterised *NRT1* genes (Table S1). Results from our analyses identified grass orthologues of *AtNRT1.2*, *AtNRT1.3* and *AtNRT1.4*. However, for the remaining five members, a lack of resolution compounded by the analysis of only a subset of the 53 *NRT1*(*PTR*) genes and the complexity of ancestral events, rendered clear orthologous relationships between dicots and monocots, unattainable.

**Figure 1:**
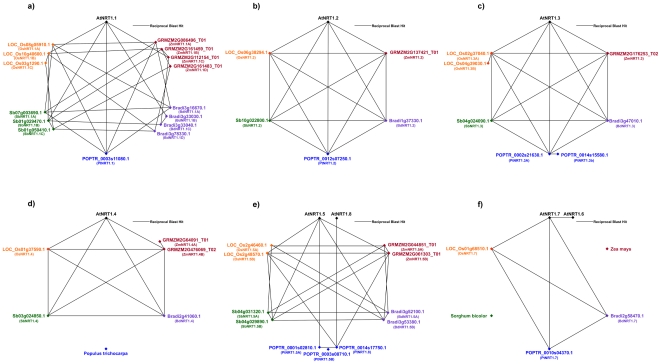
NRT1 reciprocal BLAST polygons. Reciprocal best hits are connected by black lines, for Arabidopsis (black), maize (red), *Brachypodium* (purple), poplar (blue), sorghum (green) and rice (orange). Results are depicted for (A) AtNRT1.1, (B) AtNRT1.2, (C) AtNRT1.3, (D) AtNRT1.4, (E) AtNRT1.5 and AtNRT1.8 and (F) AtNRT1.7.

RBH BLASTp E values in both forward and reverse direction for all eight *NRT1* genes was less than 10^−139^, with *AtNRT1.1* – *AtNRT1.5* and *AtNRT1.8* showing greater sequence similarity to their grass counterparts (E values ≤2×10^−159^, ≈60% to 65% sequence identity across ≥85% of the query sequence) than *AtNRT1.6* and *AtNRT1.7* (≈50% sequence identity across ≥80% of the query sequence). Furthermore, RBH analyses returned near identical hits to the grass genomes for *AtNRT1.5* and *AtNRT1.8* as well as for *AtNRT1.6* and *AtNRT1.7*. Graphical representation of the dicot – monocot RBH analysis can be seen in Figure S1. All-against-all RBH results for the *NRT1* family is illustrated in Figure 1 (A–F).

**Figure 2:**
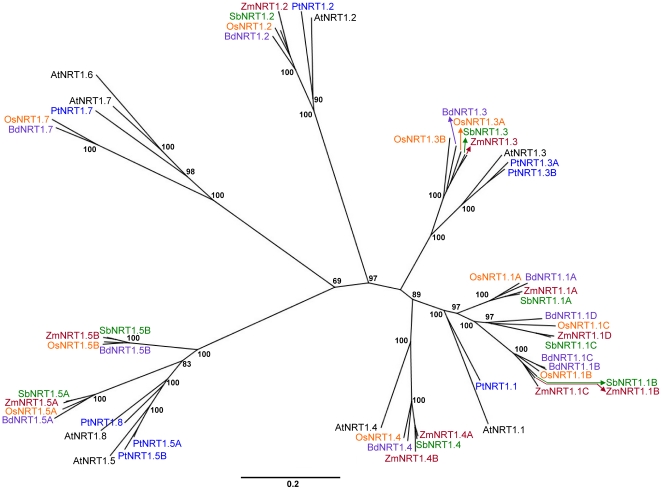
Phylogenetic relationship of the NRT1 family. Unrooted Neighbour-joining tree of NRT1 transporters in Arabidopsis (black), poplar (blue) and 4 grass species: rice (orange), sorghum (green), maize (red) and *Brachypodium* (purple). Bootstrap values from 1,000 replicates were used to estimate the confidence limits of the nodes. The scale bar represents a 0.2 estimated amino acid substitution per residue.

The phylogenetic tree of the eight Arabidopsis *NRT1* genes and their identified grass homologues (Figure 2) depicts these findings clearly. For *AtNRT1.1*, there is one gene in poplar; in the grasses, however, there are three clades of closely related *NRT1.1*-like genes all containing at least one representative from each grass species (subclade 3 has an extra *Brachypodium* and maize gene). As the genes fall into three subclades, it is likely that gene duplication events gave rise to the three groups after the dicot-monocot split. For *AtNRT1.2*, *AtNRT1.3* and *AtNRT1.4* we see a much clearer picture, all cluster with a single clade of grass genes containing at least one member from the four grass species (rice and maize have an extra representative in the 1.3 and 1.4 clade respectively). Notably, poplar has no *AtNRT1.4*-like gene. *AtNRT1.5* and *AtNRT1.8* sit together with two and one poplar orthologues respectively and branch off from the main tree with two separate but complete clades of grass genes. Due to a slightly higher dicot – monocot RBH score for 1.5 over 1.8, the grass members of these two clades were named 1.5A and 1.5B. Upon close scrutiny of this branch, we found that the grass 1.5B clade had a nearer phylogenetic neighbour (a *PTR* gene – At5g19640) even though BLAST result scores were higher for 1.5 and 1.8. Since both clades sit equidistant from *AtNRT1.5* (and neither more closely related to *AtNRT1.8*) an unambiguous assignment of orthology is not possible. Similarly, *AtNRT1.6* and *AtNRT1.7* sit together on the tree but unlike 1.5 and 1.8, 1.6 and 1.7 share one poplar orthologue and branch of the main tree with a single clade of grass genes missing both a maize and sorghum representative. Interestingly, the sorghum genome was found to contain a degraded pseudogene version that is related to *NRT1.6* and *NRT1.7*, but this transcript is likely to be non-existent as no ESTs exist in any database.

Additionally, eight monocot sequences - three maize, three sorghum and one each from rice and *Brachypodium* - were initially included in the potential orthologue list after RBH analysis but rejected after refinement and validation. These sequences were not included as they were clearly seen to be nearest neighbours to three Arabidopsis *PTR* genes (Figure S2) and this was authenticated in RBH scores.

### *NRT2* family

**Figure 3:**
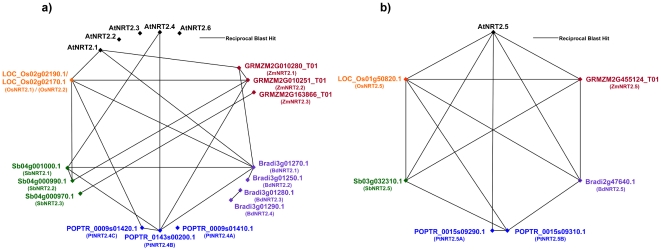
NRT2 reciprocal BLAST polygons. Reciprocal best hits are connected by black lines, for Arabidopsis (black), maize (red), *Brachypodium* (purple), poplar (blue), sorghum (green) and rice (orange). Results are depicted for (A) AtNRT2.1 or AtNRT2.2 or AtNRT2.3 or AtNRT2.4 or AtNRT2.6 and (B) AtNRT2.5.

The seven members of the *NRT2* family in Arabidopsis possessed a greater sequence similarity with each other than did the members of the *NRT1* family (Table S1). RBH analysis of the *NRT2* family returned identical results for *AtNRT2.1* – *AtNRT2.4* and *AtNRT2.6* (E values  =  zero, 62% to 72% sequence identity across ≈90% of the query sequence). Results of the best hits for *AtNRT2.5* were marginally lower (E values ranging between 10^−162^ and zero). Graphical representation of the dicot – monocot RBH analysis can be seen in Figure S1. All-against-all RBH results for the *NRT2* family is illustrated in Figure 3.

**Figure 4:**
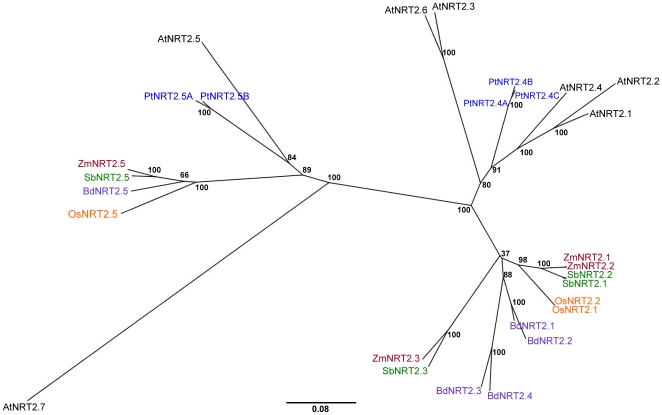
Phylogenetic relationship of the NRT2 family. Unrooted Neighbour-joining tree of NRT2 transporters in Arabidopsis (black), poplar (blue) and 4 grass species: rice (orange), sorghum (green), maize (red) and *Brachypodium* (purple). Bootstrap values from 1,000 replicates were used to estimate the confidence limits of the nodes. The scale bar represents a 0.08 estimated amino acid substitution per residue.

Results from analyses on the *NRT2* family painted a particularly interesting picture, exclusive of *NRT2.5*, the grass *NRT2* genes sit entirely separate on the phylogenetic tree from the Arabidopsis *NRT2* genes (Figure 4). Three poplar sequences (named *PtNRT2.4A*, *PtNRT2.4B* and *PtNRT2.4C* due to a higher dicot – monocot RBH score with *AtNRT2.4*) cluster with the Arabidopsis sequences suggesting that the *NRT2* genes developed primarily following the divergence of the monocots and dicots. Thus identification of a clear grass orthologues was only achieved for *AtNRT2.5*. A close investigation of the genomic localisation of these genes revealed clustering of the genes somewhat reminiscent of genes involved in disease resistance. *AtNRT2.1* and *AtNRT2.2* are neighbouring genes in opposing orientation, *AtNRT2.3* and *AtNRT2.4* are tandem repeats and *AtNRT2.6* (which peculiarly has the highest pairwise sequence similarity over all pairwise matches in the group, to *AtNRT2.3*–89%) was located on a completely separate chromosome to the others. Correspondingly, of the twelve related sequences in grass (2.1–2.4 in Figure 4), eleven are located in close proximity to another in their respective genomes. Maize has three genes related to this *NRT2* branch, two of which are closely located on chromosome 4, the other gene being located on a separate chromosome (Figure S3). Similarly, sorghum has two closely located *NRT2* genes with a third related gene located one gene to the upstream side of the closely located pair; this third related gene clusters in the phylogenetic tree with the third *NRT2* gene from maize. Rice has a similar pair of closely located *NRT2* genes (actually giving rise to the same protein sequence), while *Brachypodium* has two sets of closely located *NRT2* genes which are immediately adjacent to each other. Interestingly, the pair of *NRT2* genes found in each of the grass species is generally separated by a non-*NRT2* gene. These non-*NRT2* genes do not share any sequence similarity with one another, nor to any other functionally characterised proteins in the databases (BLASTx search against NCBI). The pairs of *NRT2* genes in the grass species may have similar function to *AtNRT2.1* and *AtNRT2.2*, although confirmation of this would require proper functional analysis. Analysis of the gene structure of the *NRT2* genes showed that the dicot *NRT2* genes all contain both exons and introns, while none of the grass *NRT2* genes contain introns (Table S2); this would indicate an ancient divergence of the members of the *NRT2* gene family.

There is one distinct *NRT2.5*-like gene in each grass genome; poplar, however, has two copies. There are no *NRT2.7*-like genes in any of the grass genomes, or in poplar. *AtNRT2.7* is the most diverged of all the *NRT2* sequences.

### *NRT3* family

The NRT3 family in Arabidopsis contains two members, *AtNRT3.1* and *AtNRT3.2*. These genes are not NO_3_^−^ transporters, but have been shown to be necessary for NO_3_^−^ transport through interaction with other NRT2 transporters. The grass genomes were analysed for orthologues to both of these Arabidopsis *NRT3* genes.

Genome analysis revealed the *NRT3* family is best represented by individual splice forms. For *NRT3.2* three splice variants have been predicted giving rise to two different amino acid sequences (www.arabidopis.org, TAIR Acc# At4G24730) (Figure S4 and S5). In comparison to the 443 aa long protein encoded by the longest splice form (At4G24730.1, hereafter referred to as *AtNRT3.2*), the proteins translated from the other two splice forms (At4G24730.2 and At4G24730.3, which will be referred to as NRT3.2SF2/3) are 311aa in length, the first 281 aa being identical with that of the longest splice form. Furthermore, there is evidence (NCBI Acc# DQ492237) for another splice product from this locus, which gives rise to a protein of 209 aa overlapping with the C-terminal half of the longest splice form. This protein sequence (referred to here as NRT3.2CT) was described and compared to NRT3.1 by Okamoto et al [20], revealing that the two proteins share 61% amino acid sequence identity.

When the protein sequence of At4G24730.1 was used in BLAST searches it became apparent that in the other species in this study the N- and C terminal halves of At4G24730.1 are coded for by two distinct genes. Consequently, it was decided that the protein sequences of NRT3.2SF2/3 and NRT3.2CT would be used separately for orthology searches. Notably, three splice forms have been predicted for *NRT3.1* but all three of these translate into the same protein (http://arabidopsis.org/servlets/TairObject?type=gene&name=AT5G50200.3).

**Figure 5:**
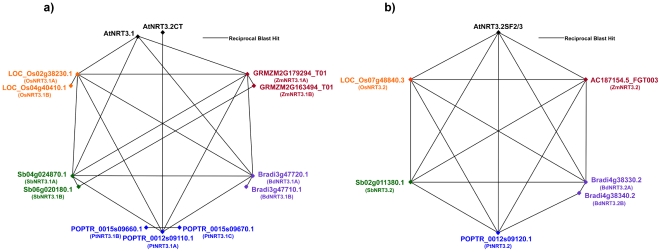
NRT3 reciprocal BLAST polygons. Reciprocal best hits are connected by black lines, for Arabidopsis (black), maize (red), *Brachypodium* (purple), poplar (blue), sorghum (green) and rice (orange). Results are depicted for (A) AtNRT3.1 or AtNRT3.2CT and (B) AtNRT3.2 or AtNRT3.2SF2/3.

**Figure 6:**
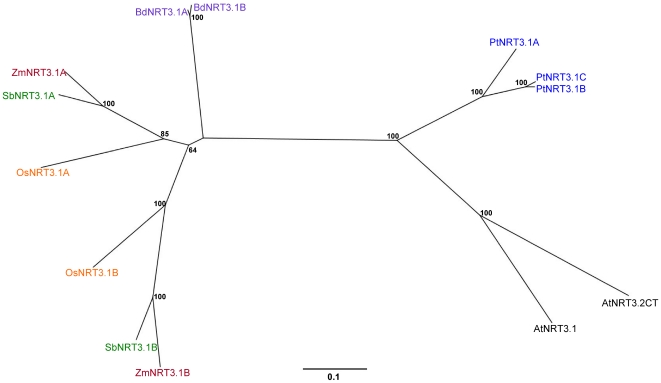
Phylogenetic relationship of the NRT3.1 or 3.2CT family. Unrooted Neighbour-joining tree of NRT3.1 or 3.2CT family in Arabidopsis (black), poplar (blue) and 4 grass species: rice (orange), sorghum (green), maize (red) and *Brachypodium* (purple). Bootstrap values from 1,000 replicates were used to estimate the confidence limits of the nodes. The scale bar represents a 0.1 estimated amino acid substitution per residue.

The results from RBH analysis were the same for both AtNRT3.1 and AtNRT3.2CT, identifying a pair of co-orthologous genes in maize, sorghum and rice and a tandem repeat in *Brachypodium* (Figure S1). Interestingly, poplar has three representative homologues of AtNRT3.1/3.2CT. BLAST E-values in both forward and reverse direction ranged between 10^−27^ and 10^−32^, with sequence identities ranging from 45% to 52% across 66% to 74% of the query sequence for AtNRT3.1 and ≈41% across ≈85% of the query sequence for AtNRT3.2CT (Graphical representation of the dicot – monocot RBH analysis can be seen in Figure S1). Closer investigation of the BLAST results to maize, rice and sorghum revealed a pattern; the genomic organisation of all these genes was well conserved between species. We postulate that either; a duplication event took place before the monocot – dicot split giving rise to co-orthologous pairs of genes of which one was eventually lost in Arabidopsis, or that, recent but separate duplication events have occurred in both the grass species and poplar, independent to Arabidopsis. RBH results for AtNRT3.1 and AtNRT3.2CT are illustrated in Figure 5A. Figure 6 shows the phylogenetic relationship of these protein sequences.

**Figure 7:**
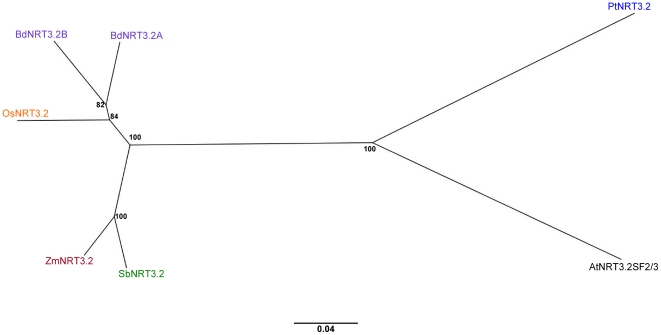
Phylogenetic relationship of the NRT3.2SF2/3 family. Unrooted Neighbour-joining tree of NRT3.2SF2/3 family in Arabidopsis (black), poplar (blue) and 4 grass species: rice (orange), sorghum (green), maize (red) and *Brachypodium* (purple). Bootstrap values from 1,000 replicates were used to estimate the confidence limits of the nodes. The scale bar represents a 0.04 estimated amino acid substitution per residue.

Similarly, AtNRT3.2 and AtNRT3.2SF2/3 produced the same RBH results, identifying a single NRT3 orthologue in maize, sorghum and rice and a tandem repeat in *Brachypodium* (Figure S1). BLAST E values in both forward and reverse direction ranged from 10^−119^ to 10^−130^, scoring ≈67% sequence identity. Alignment length differed between the two RBH analyses and was shown to be higher for NRT3.2SF2/3 (≈98%) compared with NRT3.2 (≈68%) (Graphical representation of the dicot – monocot RBH analysis can be seen in Figure S1). This difference can be explained by the fact that all BLAST hits landed within the aligned section between AtNRT3.2 and AtNRT3.2SF2/3 and never in the extended region of the former. For this reason, all subsequent investigations with respect to *NRT3.2* were undertaken with only the *AtNRT3.2SF2/3* splice form. Also similar to the *NRT3.1* genes, close scrutiny of the AtNRT3.2SF2/3 RBH results revealed second best hits in the forward direction which were reciprocal in reverse for both maize (GRMZM2G337128_T01) and sorghum (Sb07g024380.1), but not in rice. E values, sequence identity and alignment length for these hits were found to be just below those of the top hits (E value 10^−117^, sequence identity ≈62% and alignment length of ≈99%). Although considered noteworthy, the maize and sorghum sequences were not used in any subsequent analysis. Figure 5B shows RBH results for AtNRT3.2 and AtNRT3.2SF2/3 and Figure 7 indicates the phylogenetic relationship of these protein sequences.

**Figure 8:**
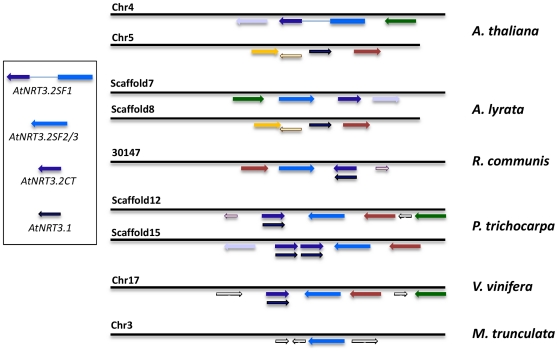
Genomic organisation of the *NRT3* genes in the dicots. Results are depicted for *Arabidopsis thaliana*, *Arabidopsis lyrata*, *Ricinus communis*, *Poplar trichocarpa*, *Vitis vinifera* and *Medicago trunculata*. The genes represented by a similar colour are reciprocal top BLAST hits. Genes are labelled with *Arabidopsis thaliana* nomenclature: *AtNRT3.1*, *AtNRT3.2SF1*, *AtNRT3.2SF2/3* and *AtNRT3.2CT*. Chromosome or scaffold numbers for each species are provided. Illustrations are not to scale.

An attempt was made to explain our finding that At4G24730.1 appeared to be a fusion of what seemed to be two distinct genes in other species. This was done by comparing the genomic organisation of the *NRT3* loci in *Arabidopsis lyrata* and in a further four dicot species each with a fully sequenced genome. As can be seen from Figure 8, only in *Arabidopsis* species is the organisation such that a di-cistronic mRNA can give rise to a fusion product through alternative splicing. In *Ricinus communis*, *Vitis vinifera* and *Poplar trichocarpa* the *NRT3.2SP2/3* gene and the *NRT3.2CT* gene are encoded on the opposite strand of the double helix, and no additional separate *NRT3.1*-like gene could be found. The exception is *Medicago trunculata* which appears to lack any *NRT3.2CT* (or *NRT3.1*) orthologues in the genome at all. This organisation, with the genes being encoded on opposite strands in direct genomic vicinity, is true also for *Carica papaya*, *Cucumis sativus*, and *Manihot esculenta* (data not shown). These results clearly indicate that the genomic organisation found in Arabidopsis may be the exception rather than the rule for dicots.

## Discussion

The purpose of this study was to determine the cereal orthologues of the characterised Arabidopsis *NRT* genes. Several studies have considered the *NRT* genes in grasses, but have resulted in some confusion, not least in the nomenclature ascribed to the various *NRT* genes identified. Much of this confusion was due, presumably, to the unavailability of fully sequenced grass genomes leading, for example, to the cloning of orthologues using degenerate primers. For instance, Lin et al [33] cloned an *NRT* gene in rice which was referred to as *OsNRT1.1* by Tsay et al [3]. This gene is part of the *NRT1*(*PTR*) family, but it is not a likely orthologue for *AtNRT1.1*. From the present analysis, and that of Tsay et al [3], it is evident that the rice genes Os08g05910 and Os10g40600 are much more likely to be the orthologues of *AtNRT1.1* than Os03g13274 as originally suggested by Lin et al [33]. Similarly, Liu et al [34] identified AY187878 as the maize orthologue of *AtNRT1.1*; however this gene does not share as high a similarity with *AtNRT1.1* as do the four genes identified in our analysis (GRMZM2G086496, GRMZM2G161483, GRMZM2G161459 and GRMZM2G112154).

The important differences which exist in *NRT* family structure between Arabidopsis and the grasses indicate that Arabidopsis may not be the best model for interpreting NO_3_^−^ transport in the grasses. The grasses have 3–4 closely related co-orthologues to AtNRT1.1. Recent work indicates that AtNRT1.1 (CHL1) may function as a nitrogen sensor [7]; should this be the case, the grasses may have more finely tuned nitrogen sensing, root tissue specific nitrogen sensors, a nitrogen sensor in the shoot tissue (depending on where these genes are expressed), or else may have a very different sensing mechanism altogether. Analysis of the NCBI Unigene database for *Oryza sativa* Build #80 (http://www.ncbi.nlm.nih.gov/UniGene/UGOrg.cgi?TAXID=4530) shows that *OsNRT1.1A* and *OsNRT1.1B* are expressed throughout the rice plant, however *OsNRT1.1A* is predominantly expressed in the root and is expressed more highly than *OsNRT1.1B*. This indicates that at least two genes potentially fill the same functional role of *AtNRT1.1* in grasses. Conversely, the grass genomes lack certain *NRT* genes that have been characterised in Arabidopsis. Our analysis reveals that the *Brachypodium* and rice genomes contain only one protein similar to *AtNRT1.7* and *AtNRT1.6*, or no orthologues in the case of maize and sorghum. AtNRT1.6 appears to be involved in transport of NO_3_^−^ from the maternal tissue to the developing embryo [13] and AtNRT1.7 plays a role in the remobilisation of NO_3_^−^ from the older leaves [14]. Again analysis of the NCBI Unigene database for *Oryza sativa* Build #80 shows *OsNRT1.7* is expressed in flower, seed and panicle, perhaps indicating that NRT1.7 in the grasses fills a similar functional role to AtNRT1.6. Whether the proposed long distance NO_3_^−^ transport functions of the AtNRT1.5 [12] and AtNRT1.8 [15] genes are indeed carried out by their closest grass homologues (NRT1.5A and NRT1.5B) or whether the grasses employ different genes remains to be investigated. The NCBI Unigene database indicates that *OsNRT1.5A* is expressed predominantly in root and panicle, but also in stem, leaf and flower. *OsNRT1.5B* was mostly expressed in panicle, but also flower and stem.

Perhaps the most obvious difference between the grasses and Arabidopsis lies in the structure of the *NRT2* gene family. The presence of closely located *NRT2* genes is reminiscent of the *AtNRT2.1*/*AtNRT2.2* cluster in Arabidopsis and functional analysis of these genes may show similar function between the *NRT2* gene clusters. However, with extra duplication in *Brachypodium*, for example, it will be interesting to identify the functional role played by each of the repeats. Since the *NRT2* genes in the grasses lack an intron, it would appear that development of the *NRT2* family occurred following the split between the dicots and monocots. It is possible that when they diverged from primitive dicots, the early monocots lost the *NRT2* intron(s) whilst the dicots retained the intron(s). However, determination of the development of the *NRT2* gene family in plants requires significant further analysis including the genomes of species all along the evolutionary tree, especially other monocots and will need to wait until more genomes are fully sequenced. The *NRT2* family is part of the Major Facilitator Superfamily (MFS); of interest, therefore, would be an investigation of the other gene families in this superfamily to establish whether they show the same dichotomy in exon/intron structure. The grass genomes do not contain an *AtNRT2.7* orthologue. Since the function of this gene is to load NO_3_^−^ into the seeds of Arabidopsis [18], again this would indicate a possible difference in the way in which grasses load NO_3_^−^ into seeds and embryos (similarly to the lack of AtNRT1.6 described above). Further analysis is required to determine whether the grass *NRT2* genes have a similar function to that of the Arabidopsis *NRT2* genes, or whether their evolutionary divergence also results in a divergence in function. Should there be a divergence in function the isolation of any common protein motifs or sequence differences that separate the dicot and grass *NRT2*s may provide important structure/function information of value in guiding biotechnological approaches to improving NO_3_^−^ transport in plants. Although not included in our full analysis (since the genome has not been fully sequenced) the four barley (*Hordeum vulgare*) *NRT2*s [35], [36] group with the two pairs of *Brachypodium NRT2* genes (*BdNRT2.1*/*BdNRT2.2* and *BdNRT2.3*/*BdNRT2.4*) (Figure S6); consequently, the functional analysis of these genes provides little insight into the potential function of the *NRT2* genes from the other grasses except to indicate the cereal NRT2 transporters likely mediate high affinity NO_3_^−^ transport as the barley NRT2 transporters do.

Our analysis of the NRT3 family also indicates there may be fundamental differences between Arabidopsis and the grasses indicative of unique evolutionary events. Of particular importance, the significance of the fusion of the *AtNRT3.2SF2/3* gene with the *AtNRT3.1CT* gene in Arabidopsis compared with the way this protein interacts with the high affinity NRT2 transporters in grasses remains unknown. It will be interesting to determine whether the *AtNRT3.2SF2/3* orthologues in the grasses are involved in NO_3_^−^ transport. In barley (*Hordeum vulgare*), three *NRT3* genes (*NAR2.1*, *NAR2.2* and *NAR2.3*) have been identified [37], all of which are similar to the *AtNRT3.1* or *AtNRT3.2CT* genes (Figure S7). It remains to be seen whether or not the *NRT3* genes identified in this study play a functional role similar to that of the barley orthologues.

To assist future research we have developed a nomenclature for grass *NRT* genes (Table 1). Without functional characterisation of the grass NRT orthologues it is difficult to determine which grass orthologue will have similar function to a given Arabidopsis gene, especially in the case of genes with multiple candidates (e.g. *NRT1.1*). We have attempted to take this issue into account by naming all potential co-orthologues A, B, C, etc. However, in the case of the *NRT2* gene family where dicot and grass genes do not cluster together in the phylogenetic tree, this approach becomes problematic. Therefore, we named family members *NRT2.1*, *NRT2.2*, etc, despite the fact that a grass NRT2.1 may not share a functional role with AtNRT2.1.

**Table 1:** Summary of the gene identifiers and new *NRT* nomenclature for *NRT1*, *2* and *3* genes in Arabidopsis, poplar, rice, maize, sorghum and *Brachypodium*.

*Arabidopsis thaliana*		*Populus trichocarpa*		*Oryza sativa*		*Zea mays*		*Sorghum bicolor*		*Brachypodium distachyon*	
Symbol	TAIR ID	Symbol	JGI ID	Symbol	MSU ID	Symbol	Maizesequences.orgID	Symbol	JGI ID	Symbol	Brachypodium.orgID
AtNRT1.1	AT1G12110	PtNRT1.1	POPTR_0003s11080.1	OsNRT1.1A OsNRT1.1B OsNRT1.1C	LOC_Os08g05910.1 LOC_Os10g40600.1 LOC_Os03g01290.1	ZmNRT1.1A ZmNRT1.1B ZmNRT1.1C ZmNRT1.1D	GRMZM2G086496_P01 GRMZM2G161459_P02 GRMZM2G112154_P01 GRMZM2G161483_P01	SbNRT1.1A SbNRT1.1B SbNRT1.1C	Sb07g003690.1 Sb01g029470.1 Sb01g050410.1	BdNRT1.1A BdNRT1.1B BdNRT1.1C BdNRT1.1D	Bradi3g16670.1 Bradi3g33030.1 Bradi3g33040.1 Bradi1g78330.1
AtNRT1.2	AT1G69850	PtNRT1.2	POPTR_0012s07250.1	OsNRT1.2	LOC_Os06g38294.1	ZmNRT1.2	GRMZM2G137421_P01	SbNRT1.2	Sb10g022800.1	BdNRT1.2	Bradi1g37330.1
AtNRT1.3	AT3G21670	PtNRT1.3A PtNRT1.3B	POPTR_0002s21630.1 POPTR_0014s15580.1	OsNRT1.3A OsNRT1.3B	LOC_Os02g37040.1 LOC_Os04g39030.1	ZmNRT1.3	GRMZM2G176253_P02	SbNRT1.3	Sb04g024090.1	BdNRT1.3	Bradi3g47010.1
AtNRT1.4	AT2G26690	N/A	N/A	OsNRT1.4	LOC_Os01g37590.1	ZmNRT1.4A ZmNRT1.4B	GRMZM2G064091_P01 GRMZM2G476069_P01	SbNRT1.4	Sb03g024850.1	BdNRT1.4	Bradi2g41060.1
AtNRT1.5	AT1G32450	PtNRT1.5A PtNRT1.5B	POPTR_0001s02810.1 POPTR_0003s08710.1	OsNRT1.5A OsNRT1.5B	LOC_Os02g46460.1 LOC_Os02g48570.1	ZmNRT1.5A ZmNRT1.5B	GRMZM2G044851_P01 GRMZM2G061303_P01	SbNRT1.5A SbNRT1.5B	Sb04g031320.1 Sb04g029890.1	BdNRT1.5A BdNRT1.5B	Bradi3g52100.1 Bradi3g53380.1
AtNRT1.6	AT1G27080	N/A	N/A	N/A	N/A	N/A	N/A	N/A	N/A	N/A	N/A
AtNRT1.7	AT1G69870	PtNRT1.7	POPTR_0010s04370.1	OsNRT1.7	LOC_Os01g68510.1	N/A	N/A	N/A	N/A	BdNRT1.7	Bradi2g58470.1
AtNRT1.8	AT4G21680	PtNRT1.8	POPTR_0014s17750.1	N/A	N/A	N/A	N/A	N/A	N/A	N/A	N/A
AtNRT2.1 AtNRT2.2 AtNRT2.3 AtNRT2.4 AtNRT2.6	AT1G08090 AT1G08100 AT5G60780 AT5G60770 AT3G45060	PtNRT2.4A PtNRT2.4B PtNRT2.4C	POPTR_0009s01410.1 POPTR_0143s00200.1 POPTR_0009s01420.1	OsNRT2.1 OsNRT2.2	LOC_Os02g02190.1 LOC_Os02g02170.1	ZmNRT2.1 ZmNRT2.2 ZmNRT2.3	GRMZM2G010280_P01 GRMZM2G010251_P01 GRMZM2G163866_P01	SbNRT2.1 SbNRT2.2 SbNRT2.3	Sb04g001000.1 Sb04g000990.1 Sb04g000970.1	BdNRT2.1 BdNRT2.2 BdNRT2.3 BdNRT2.4	Bradi3g01270.1 Bradi3g01250.1 Bradi3g01280.1 Bradi3g01290.1
AtNRT2.5	AT1G12940	PtNRT2.5A PtNRT2.5B	POPTR_0015s09290.1 POPTR_0015s09310.1	OsNRT2.5	LOC_Os01g50820.1	ZmNRT2.5	GRMZM2G455124_P01	SbNRT2.5	Sb03g032310.1	BdNRT2.5	Bradi2g47640.1
AtNRT2.7	AT5G14570	N/A	N/A	N/A	N/A	N/A	N/A	N/A	N/A	N/A	N/A
AtNRT3.1 AtNRT3.2CT	AT5G50200 DQ492237*	PtNRT3.1A PtNRT3.1B PtNRT3.1C	POPTR_0012s09110.1 POPTR_0015s09660.1 POPTR_0015s09670.1	OsNRT3.1A OsNRT3.1B	LOC_Os02g38230.1 LOC_Os04g40410.1	ZmNRT3.1A ZmNRT3.1B	GRMZM2G179294_P01 GRMZM2G163494_P01	SbNRT3.1A SbNRT3.1B	Sb04g024870.1 Sb06g020180.1	BdNRT3.1A BdNRT3.1B	Bradi3g47720.1 Bradi3g47710.1
AtNRT3.2SF2/3	AT4G24730.2/3	PtNRT3.2	POPTR_0012s09120.1	OsNRT3.2	LOC_Os07g48840.3	ZmNRT3.2	AC187154.5_FGP003	SbNRT3.2	Sb02g011380.2	BdNRT3.2A BdNRT3.2B	Bradi4g38330.2 Bradi4g38340.2

*GenBank Identifer.

In conclusion, the present analysis of the *NRT* gene families in Arabidopsis and the grasses has revealed some striking differences in gene family structure. Important questions about the evolution of NRT transporters in plants and, significantly, about the suitability of Arabidopsis as a model for NO_3_^−^ transport in the grasses have also been posed. With the current exponential increase in the availability of molecular genetic resources for cereal crop plants it appears likely that the relevance of Arabidopsis research will decline. This analysis provides a framework for future studies of NO_3_^−^ transporters and transport in the grasses, and potentially will guide strategies for improvement of NUE in cereal species through genetic manipulation of the *NRT* genes.

## Materials and Methods

### Sequences and Databases

DNA and amino acid sequences of 17 *AtNRT* family members (Table S1.) were retrieved from TAIR (The Arabidopsis Information Resource), *Arabidopsis thaliana* genome annotation database release 9 (http://www.arabidopsis.org/). The complete database of predicted amino acid sequences from *Arabidopsis thaliana* and *Populus trichocarpa* as well as from four monocot species; *Zea mays*, *Oryza sativa*, *Brachypodium distachyon* and *Sorghum bicolor* were downloaded from public databases (Table S3).

### Bioinformatics

#### Identification of homologues

Identification of homologues was based primarily on sequence similarity between the 17 Arabidopsis NRTs and the predicted amino acid sequences of the four above mentioned monocots (maize, rice, *Brachypodium* and sorghum). This was achieved by using BLASTp, standalone version 2.2.21 [38], [39] and a modified reciprocal best hit (RBH) approach [28], [29]. In brief, BLAST searches were performed which queried the set of 17 AtNRT protein sequences against each of the poplar/monocot databases in a pairwise manner (forward BLAST). Following this and deviating from the standard method, the top ‘cluster of best hits’ returned from each pairwise forward BLAST (based on E-value and sorted by score) was then used as queries in subsequent BLAST searches against the Arabidopsis database (reverse BLAST). Proteins from the reverse BLAST that returned as one of their best hits the original query protein from the forward BLAST (the relevant AtNRT), were then selected for further evaluation as homologues. For all forward and reverse BLASTp searches, E value cut-off was set to 1E-20 (-e 1E-20), output was set to tabular (-m 8), all other parameters were left as default. The aforementioned deviation introduced to the method used in this study was made in an attempt to resolve nearest phylogenetic neighbour and paralogy shortcomings when using BLAST and the strict RBH approach to identify homologues between species [30]. The list of homologues was then refined by removal of those candidates not specifically related to the AtNRTs of interest (Table S4). This was achieved via manual inspection of multiple sequence alignments and their corresponding trees. Throughout the analyses all splice variants of all identified homologues accepted for further analysis were used in subsequent rounds of RBH (eleven candidate homologues were found to possess splice variants, see Table S1). However, only the one member with the longest protein sequence from each splice variant group was used to build trees.

The remaining candidates were then used in all-against-all rounds of RBH analysis between the four monocot sequence databases. The RBH approach and criteria used for the original monocot to dicot search, as described above, was again applied here.

#### Multiple Sequence Alignment and Tree building

The RBH obtained sequences for NRT1, were aligned by MAFFT version 6.240 using the L-INS-I method with associated default parameters at (http://align.genome.jp/mafft/) [40] and manual editing, for NRT2, 3.1 and 3.2 by T-coffee::Advanced [41] using the server at the Swiss Institute for Bioinformatics, pairwise method set to ‘best_pair4prot’, multiple method set to ‘mafft_msa’ and default for all other parameters (http://tcoffee.vital-it.ch/). Trees were built using various programs of the Phylogenetic Interference Package (PHYLIP) 3.63 (J. Felsenstein, http://evolution.genetics.washington.edu/phylip.html). One thousand bootstrap datasets were generated with SEQBOOT to estimate the confidence limits of nodes. Protein distance matrices were calculated with PROTDIST using the PMB model [42]. Trees were generated with WEIGHBOR [43], and the majority rule consensus tree was generated by CONSENSE (default method used ‘Majority rule (extended)’ >50%). Trees were visualized using the software *Geneious v4.8* (Biomatters Ltd, New Zealand, http://www.geneious.com/). Information on genome organisation was obtained from the Phytozome (www.phytozome.org) and TAIR.

## Supporting Information

Figure S1 **AtNRT1, 2 and 3 reciprocal BLASTp results.** Depiction of forward and reverse reciprocal BLASTs is provided for (A) poplar, (B) *Brachypodium*, (C) maize, (D) sorghum and (E) rice. BLASTp hits of equal e‐value in forward and reverse directions are depicted as forward facing and reverse facing arrows respectively. The colour code of all arrows represents the order of hits, as returned by the BLAST program, first best hit (red), second best hit (blue), third best hit (orange), fourth best hit (green), fifth best hit (black). Truncated arrows indicate a minor drop in the e‐value score between hits. Also coded are the gene accession numbers; an asterisk (*) indicates the presence of alternative splice forms all scoring identical BLASTp results, an upward facing arrow head (^∧^) indicates a tandem repeat with identical BLAST score, red accessions indicate neighbouring genes in opposing orientation with identical BLAST scores and blue accessions indicate genes in close proximity to each other with identical BLAST scores.
(TIF)

Figure S2 **Phylogenetic relationship of potential grass PTR transporters orthologues to AtNRT1.6 and AtNRT1.7.** Unrooted Neighbour‐joining tree of NRT1 transporters in Arabidopsis (black), poplar (blue) and 4 grass species: rice (orange), sorghum (green), maize (red) and *Brachypodium* (purple). Highlighted in the box are the three closest PTR homologues to AtNRT1.6 and AtNRT1.7 (AT1G18880, AT3G47960 and AT5G62680) as well as orthologous grass PTR transporters (all in brown). This figure provides rationale for exclusion of grass transporters (brown) as orthologues to AtNRT1.6 and AtNRT1.7. Bootstrap values from 1,000 replicates were used to estimate the confidence limits of the nodes. The scale bar represents a 0.2 estimated amino acid substitution per residue.
(TIF)

Figure S3 **Conservation of closely localised** ***NRT2*** **genes.** Gene identifiers and chromosome number are provided for *NRT2* genes (red) for (A) Arabidopsis, (B) poplar, (C) rice, (D) *Brachypodium*, (E) maize and (F) sorghum. Sorghum has a third closely localised *NRT2* gene (blue). Also depicted are non‐NRT2 genes (black) between NRT2 genes. Illustrations are not to scale.
(TIF)

Figure S4 **Depiction of AtNRT3 genes.** Schematic of the AtNRT3.2 locus (AT4G24730) from TAIR 9 GBrowse (http://gbrowse.arabidopsis.org/). Represented are chromosome, BAC, locus, gene models from TAIR 8 and TAIR 9 and cDNA details.
(TIF)

Figure S5 **Alignment of AtNRT3 proteins.** Proteins included are AtNRT3.1 and AtNRT3.2CT described previously Okamoto et al [20] and the new versions identified in TAIR9 (AtNRT3.2SF1 AtNRT3.2SF2 and AtNRT3.2SF3). Colour scheme for residue similarity (letter/background): black/white – non similar; blue/light blue – conservative; black/green – block of similar residues; red/yellow – identical; and green/white – weakly similar. Gene identifiers are provided in brackets. Refer to Figure 7 for genomic organisation of Arabidopsis genes.
(TIF)

Figure S6 **Phylogenetic relationship of the NRT2 family including barley family members.** Unrooted Neighbour‐joining tree of NRT2 transporters in Arabidopsis (black), poplar (blue) and 5 grass species: rice (orange), sorghum (green), maize (red), *Brachypodium* (purple) and barley (brown). The four barley members include *HvNRT2.1* (HVU34198), *HvNRT2.2* (HVU34290), *HvNRT2.3* (AF091115) and *HvNRT2.4* (AF091116). Bootstrap values from 1,000 replicates were used to estimate the confidence limits of the nodes. The scale bar represents a 0.08 estimated amino acid substitution per residue.
(TIF)

Figure S7 **Phylogenetic relationship of the NRT3.1 or NRT3.2CT family including barley family members.** Unrooted Neighbour‐joining tree of NRT3.1 or 3.2CT family in Arabidopsis (black), poplar (blue) and 5 grass species: rice (orange), sorghum (green), maize (red), *Brachypodium* (purple) and barley (brown). The three barley members include HvNRT3.1A (HvNAR2.1 ‐ AY253448), HvNRT3.1B (HvNAR2.2 ‐ AY253449) and HvNRT3.1C (HvNAR2.3 ‐ AY253450). Bootstrap values from 1,000 replicates were used to estimate the confidence limits of the nodes. The scale bar represents a 0.09 estimated amino acid substitution per residue.
(TIF)

Table S1 **Identifiers, annotations and family similarities for the Arabidopsis** ***NRT*** **genes described in this study.** Provided are the TAIR annotation and identifier, UniprotKB identifier and RefSeq identifier for each *NRT* gene. Amino acid length of each protein is provided as well as the amino acid identity (%) between the NRTx.1 protein and the other family members.
(XLS)

Table S2 **Comparison of the** ***NRT2*** **gene intron and exon numbers for dicots and grasses.** Gene identifiers and exon and intron numbers are provided for the dicots *Arabidopsis thaliana*, *Populus trichocarpa*, *Manihot esculenta*, *Ricinus communis* and *Vitis vinifera*; and for the grasses *Oryza sativa*, *Zea mays*, *Sorghum bicolor* and *Brachypodium distachyon*.
(XLS)

Table S3 **Web addresses for the genome databases searched during this study including** ***Arabidopsis thaliana*** **,** ***Brachypodium distachyon*** **,** ***Oryza sativa*** **,** ***Populus trichocarpa*** **,** ***Sorghum bicolor*** **and** ***Zea mays*** **.**
(XLS)

Table S4 **List of candidate homologues excluded from analysis after manual inspection of multiple sequence alignments and their corresponding trees.**
(XLS)
