# Supplementary figures and images for: Dichotomy in the NRT Gene Families of Dicots and Grass Species
Source: PLoS One. 2010 Dec 6;5(12):e15289. doi: 10.1371/journal.pone.0015289 (PMC2997785; doi:10.1371/journal.pone.0015289)

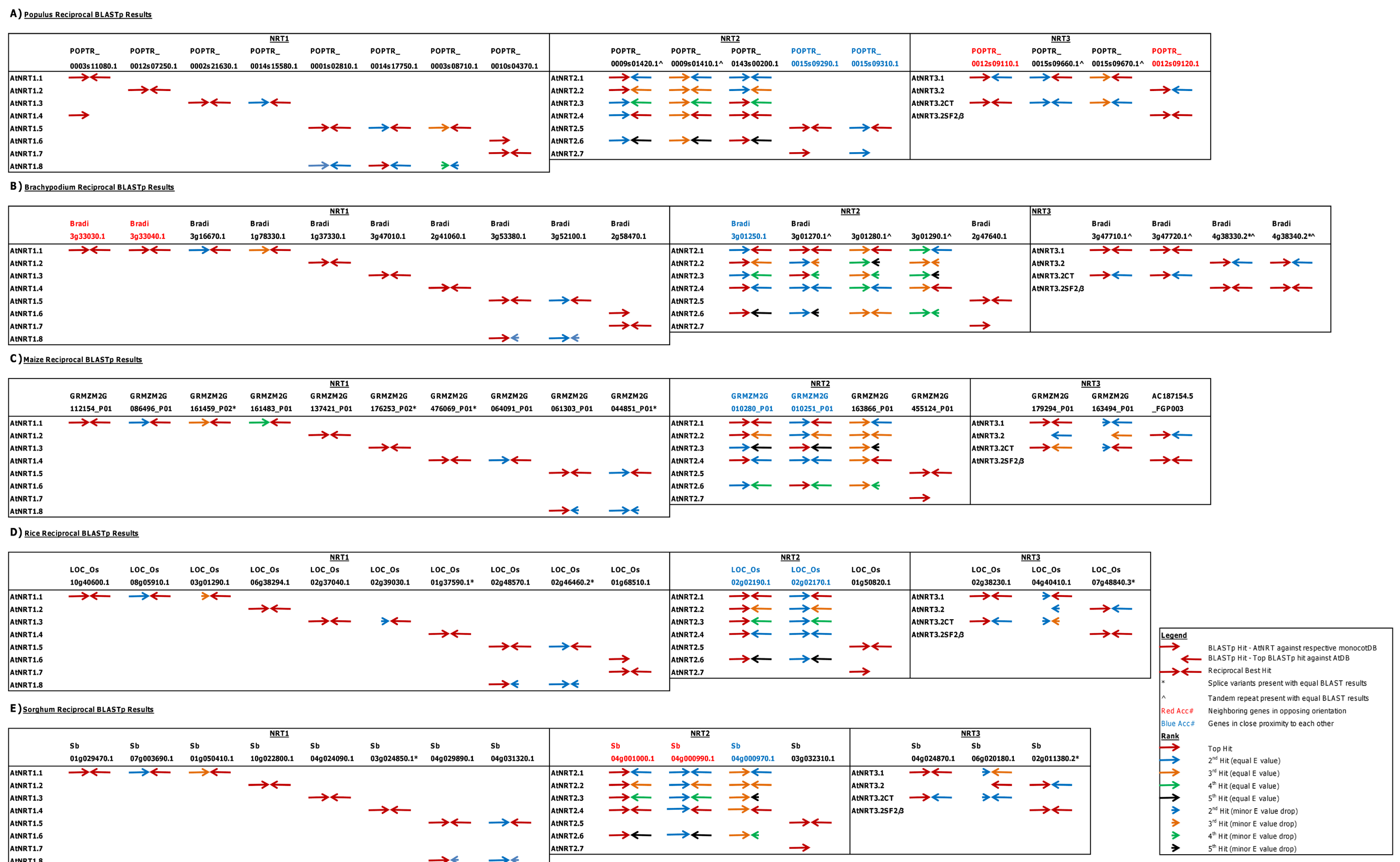

Supplement: Figure S1 — AtNRT1, 2 and 3 reciprocal BLASTp results. Depiction of forward and reverse reciprocal BLASTs is provided for (A) poplar, (B) Brachypodium, (C) maize, (D) sorghum and (E) rice. BLASTp hits of equal e‐value in forward and reverse directions are depicted as forward facing and reverse facing arrows respectively. The colour code of all arrows represents the order of hits, as returned by the BLAST program, first best hit (red), second best hit (blue), third best hit (orange), fourth best hit (green), fifth best hit (black). Truncated arrows indicate a minor drop in the e‐value score between hits. Also coded are the gene accession numbers; an asterisk (*) indicates the presence of alternative splice forms all scoring identical BLASTp results, an upward facing arrow head (∧) indicates a tandem repeat with identical BLAST score, red accessions indicate neighbouring genes in opposing orientation with identical BLAST scores and blue accessions indicate genes in close proximity to each other with identical BLAST scores. (TIF) [file pone.0015289.s001.tif]

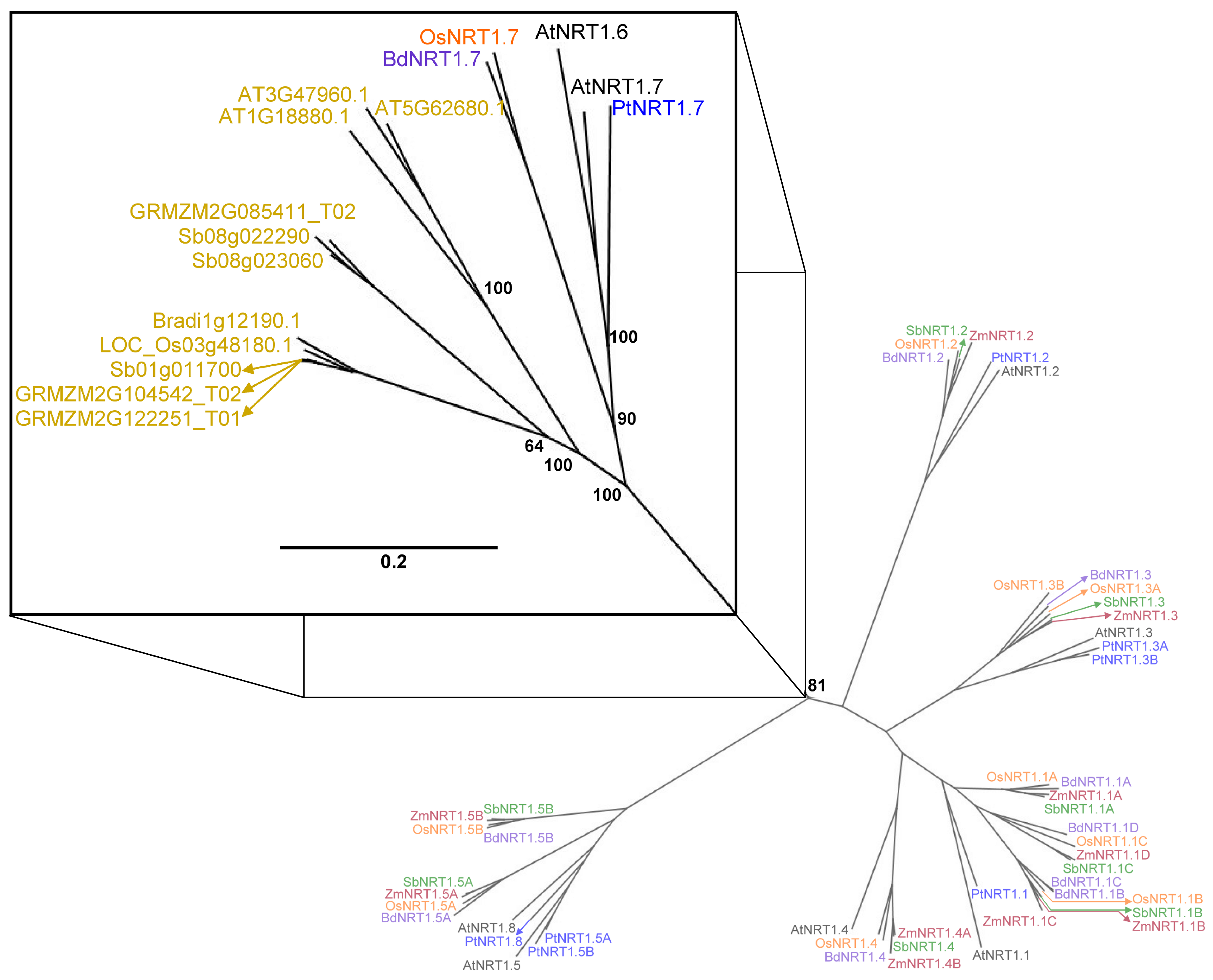

Supplement: Figure S2 — Phylogenetic relationship of potential grass PTR transporters orthologues to AtNRT1.6 and AtNRT1.7. Unrooted Neighbour‐joining tree of NRT1 transporters in Arabidopsis (black), poplar (blue) and 4 grass species: rice (orange), sorghum (green), maize (red) and Brachypodium (purple). Highlighted in the box are the three closest PTR homologues to AtNRT1.6 and AtNRT1.7 (AT1G18880, AT3G47960 and AT5G62680) as well as orthologous grass PTR transporters (all in brown). This figure provides rationale for exclusion of grass transporters (brown) as orthologues to AtNRT1.6 and AtNRT1.7. Bootstrap values from 1,000 replicates were used to estimate the confidence limits of the nodes. The scale bar represents a 0.2 estimated amino acid substitution per residue. (TIF) [file pone.0015289.s002.tif]

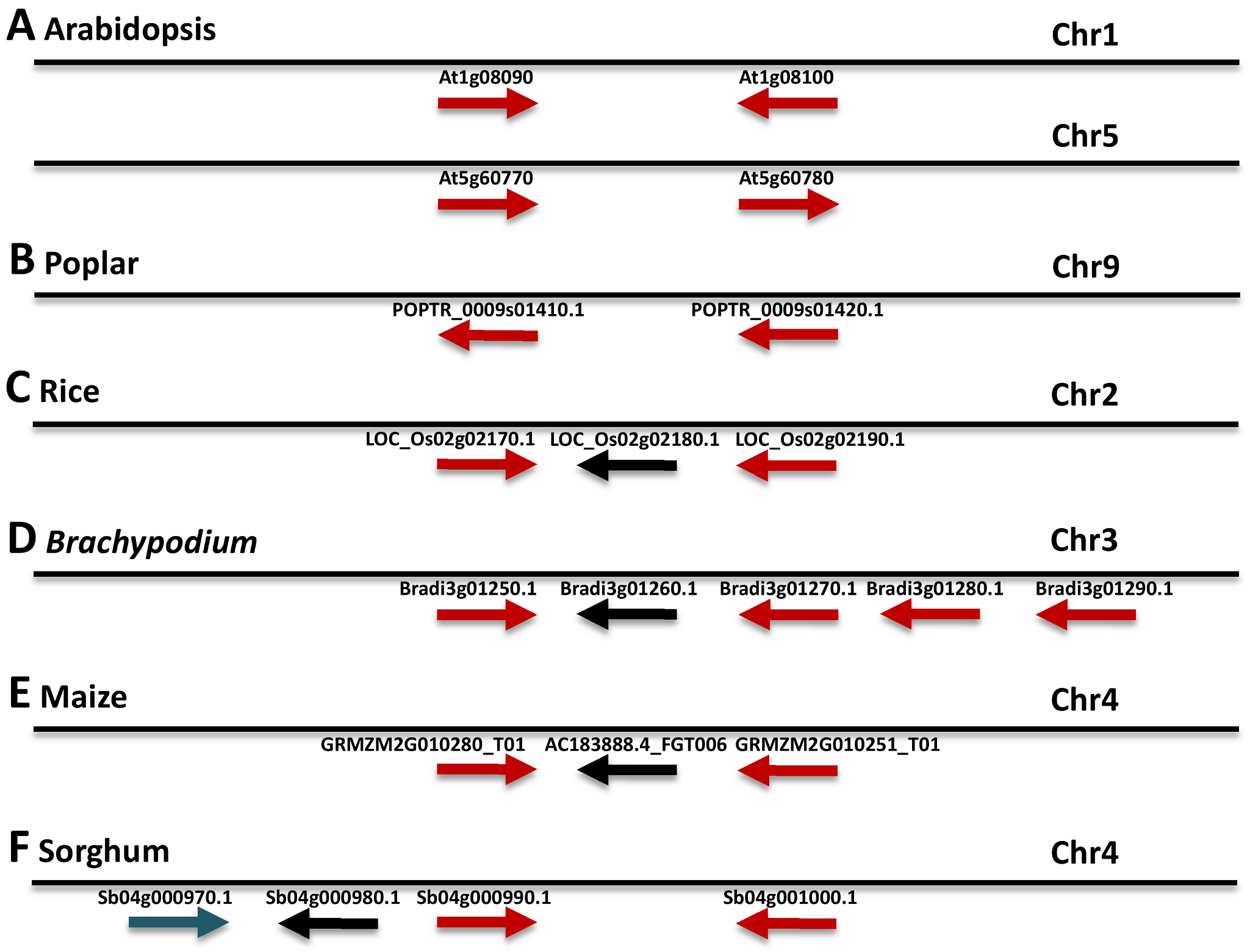

Supplement: Figure S3 — Conservation of closely localised NRT2 genes. Gene identifiers and chromosome number are provided for NRT2 genes (red) for (A) Arabidopsis, (B) poplar, (C) rice, (D) Brachypodium, (E) maize and (F) sorghum. Sorghum has a third closely localised NRT2 gene (blue). Also depicted are non‐NRT2 genes (black) between NRT2 genes. Illustrations are not to scale. (TIF) [file pone.0015289.s003.tif]

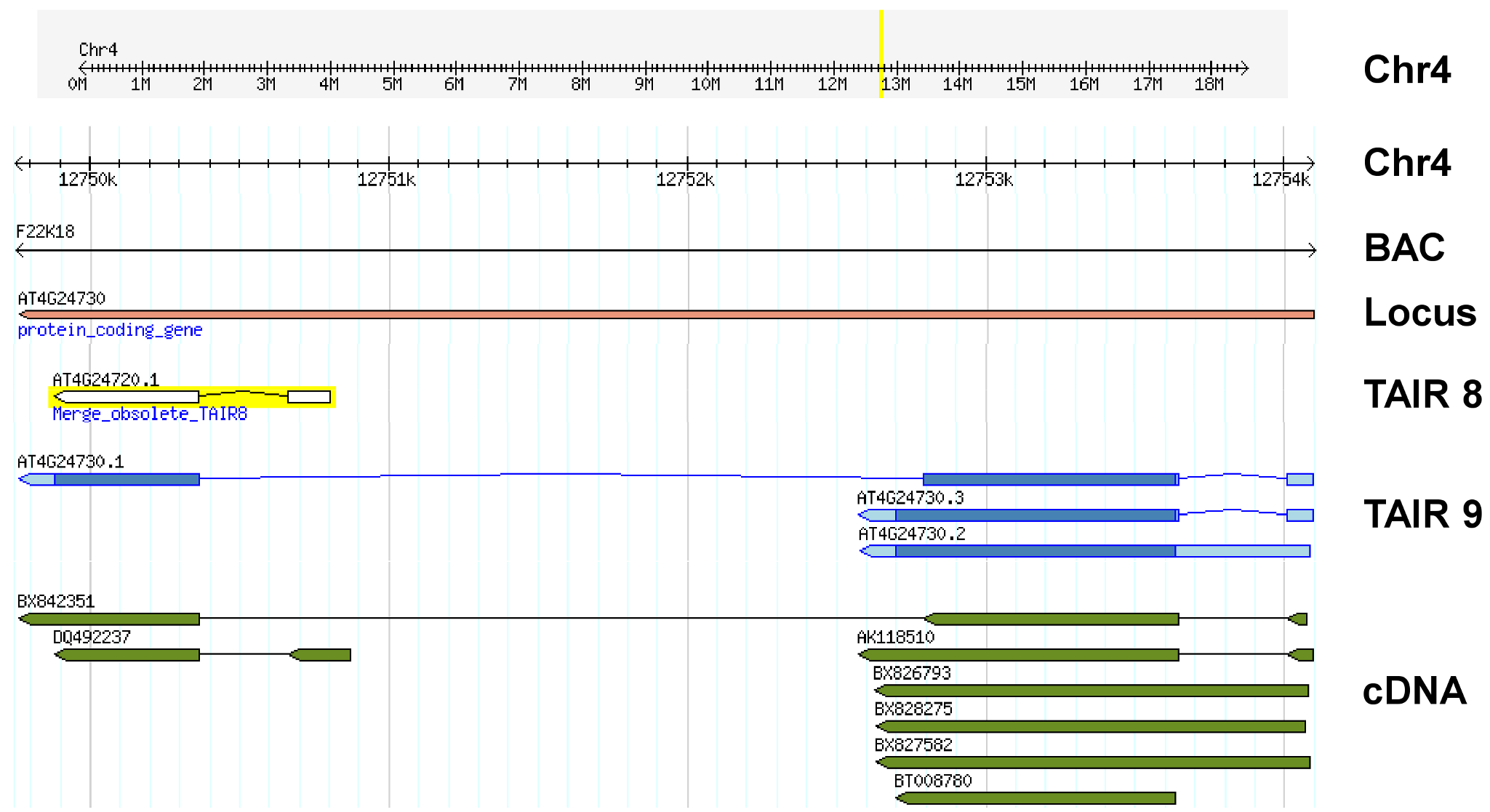

Supplement: Figure S4 — Depiction of AtNRT3 genes. Schematic of the AtNRT3.2 locus (AT4G24730) from TAIR 9 GBrowse (http://gbrowse.arabidopsis.org/). Represented are chromosome, BAC, locus, gene models from TAIR 8 and TAIR 9 and cDNA details. (TIF) [file pone.0015289.s004.tif]

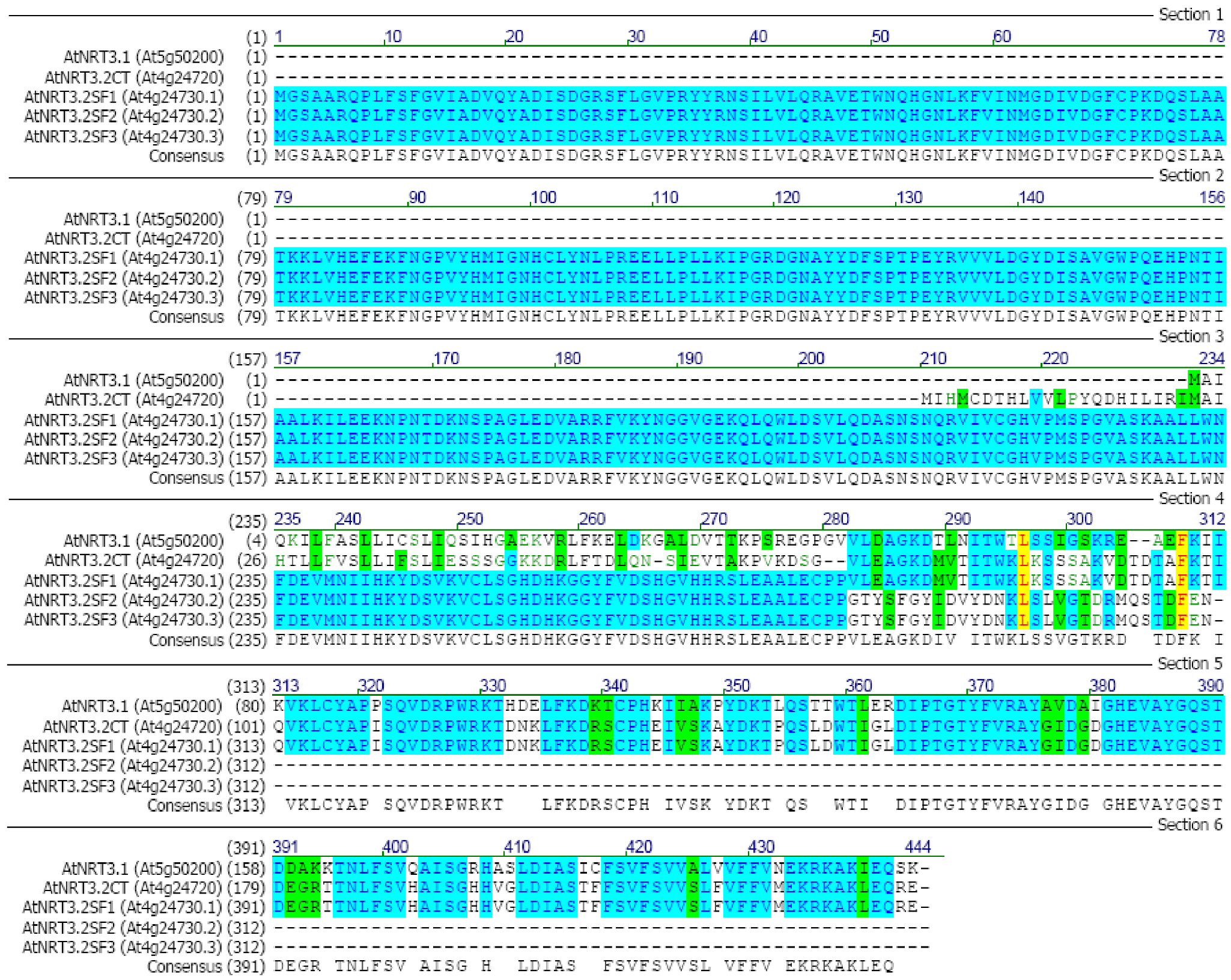

Supplement: Figure S5 — Alignment of AtNRT3 proteins. Proteins included are AtNRT3.1 and AtNRT3.2CT described previously Okamoto et al [20] and the new versions identified in TAIR9 (AtNRT3.2SF1 AtNRT3.2SF2 and AtNRT3.2SF3). Colour scheme for residue similarity (letter/background): black/white – non similar; blue/light blue – conservative; black/green – block of similar residues; red/yellow – identical; and green/white – weakly similar. Gene identifiers are provided in brackets. Refer to Figure 7 for genomic organisation of Arabidopsis genes. (TIF) [file pone.0015289.s005.tif]

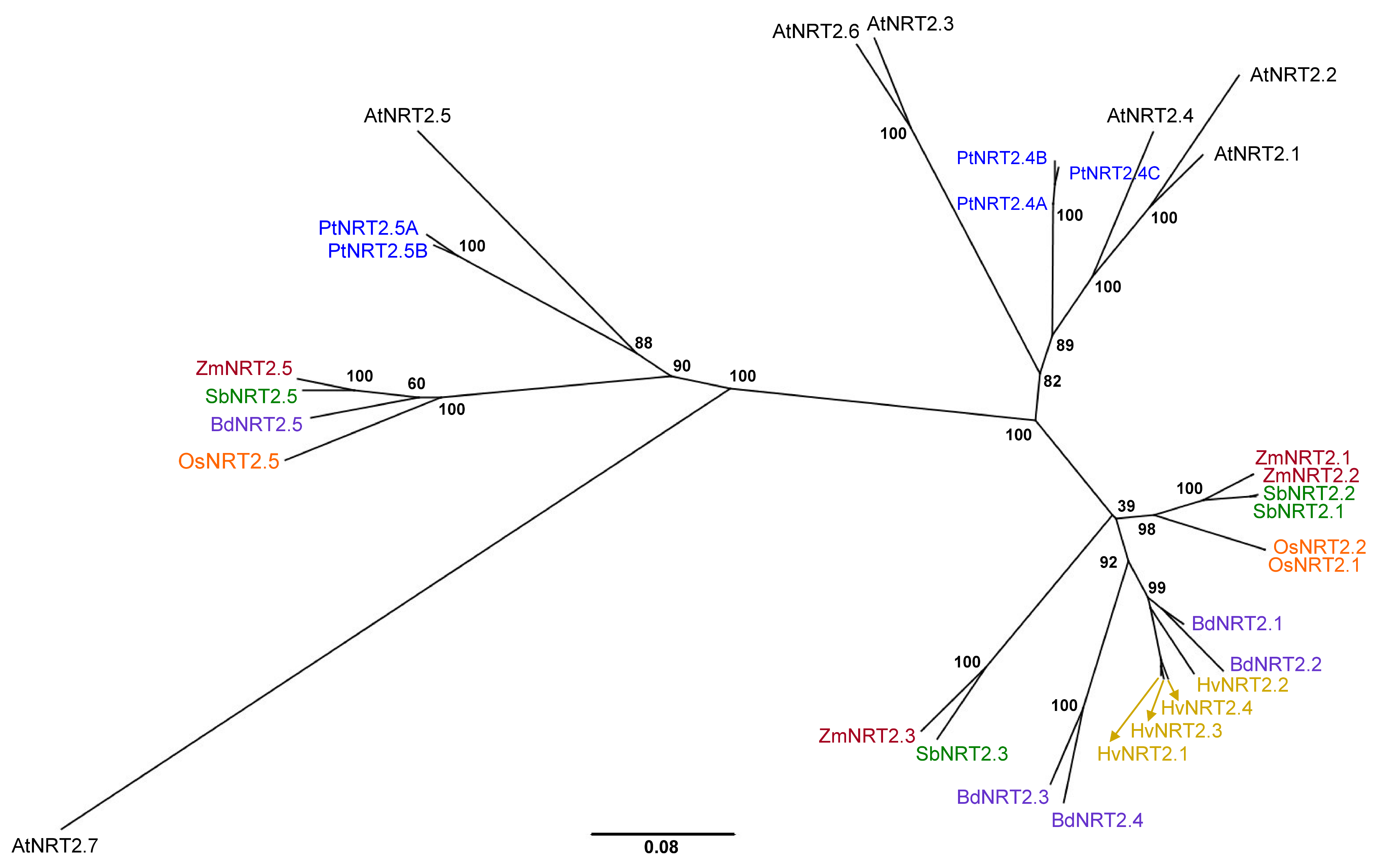

Supplement: Figure S6 — Phylogenetic relationship of the NRT2 family including barley family members. Unrooted Neighbour‐joining tree of NRT2 transporters in Arabidopsis (black), poplar (blue) and 5 grass species: rice (orange), sorghum (green), maize (red), Brachypodium (purple) and barley (brown). The four barley members include HvNRT2.1 (HVU34198), HvNRT2.2 (HVU34290), HvNRT2.3 (AF091115) and HvNRT2.4 (AF091116). Bootstrap values from 1,000 replicates were used to estimate the confidence limits of the nodes. The scale bar represents a 0.08 estimated amino acid substitution per residue. (TIF) [file pone.0015289.s006.tif]

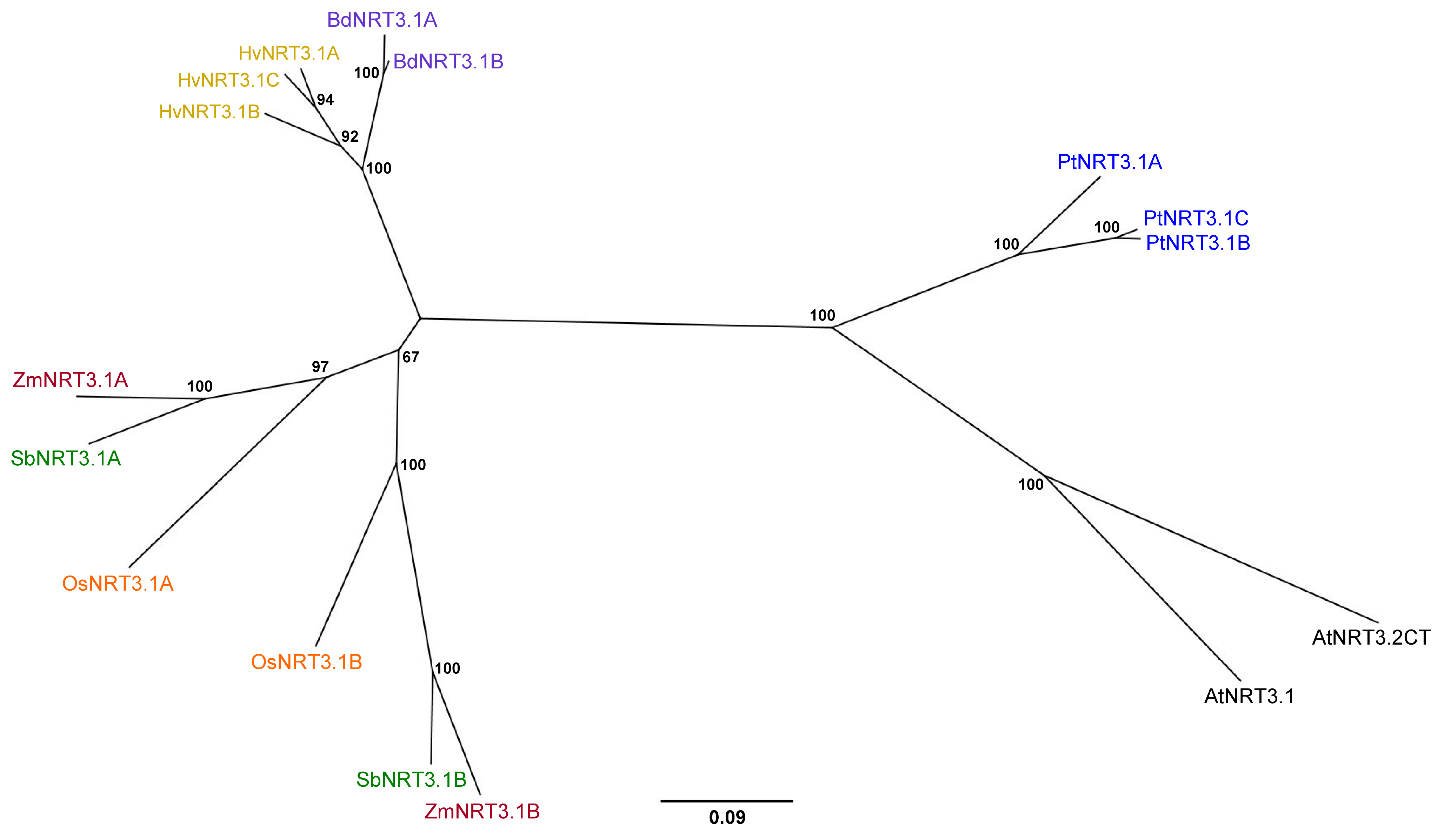

Supplement: Figure S7 — Phylogenetic relationship of the NRT3.1 or NRT3.2CT family including barley family members. Unrooted Neighbour‐joining tree of NRT3.1 or 3.2CT family in Arabidopsis (black), poplar (blue) and 5 grass species: rice (orange), sorghum (green), maize (red), Brachypodium (purple) and barley (brown). The three barley members include HvNRT3.1A (HvNAR2.1 ‐ AY253448), HvNRT3.1B (HvNAR2.2 ‐ AY253449) and HvNRT3.1C (HvNAR2.3 ‐ AY253450). Bootstrap values from 1,000 replicates were used to estimate the confidence limits of the nodes. The scale bar represents a 0.09 estimated amino acid substitution per residue. (TIF) [file pone.0015289.s007.tif]
